# Outcomes of malignancy in adults with congenital heart disease: a single center experience

**DOI:** 10.1186/s40959-022-00144-z

**Published:** 2022-11-23

**Authors:** Prashanth Venkatesh, Kimberly L. Yan, Katia Bravo-Jaimes, Eric H. Yang, Gentian Lluri

**Affiliations:** 1 Guerin Congenital Heart Program, Department of Cardiology, Cedars-Sinai Medical Center, 127 S. San Vicente Blvd, Suite A3600, Los Angeles, CA 90048 USA; 2 Department of Medicine, University of California, San Francisco, San Francisco, CA USA; 3 Division of Cardiology, Department of Medicine, Mayo Clinic Florida, Jacksonville, FL USA; 4 UCLA Cardio-Oncology Program, Division of Cardiology, Department of Medicine, University of California, Los Angeles, Los Angeles, CA USA; 5 Ahmanson/UCLA Adult Congenital Heart Disease Center, Division of Cardiology, Department of Medicine, University of California, Los Angeles, Los Angeles, CA USA

**Keywords:** Adult congenital heart disease, Cardio-oncology, Incidence, Long-term outcomes, Cardiotoxicity

## Abstract

**Background:**

Malignancy is known to be a major cause of death in adult congenital heart disease (ACHD). However, data regarding cardiovascular and cancer-related outcomes in ACHD are lacking.

**Methods:**

We conducted a retrospective single-center cohort study comprising patients with ACHD and malignancy. The primary outcome was all-cause mortality. Key secondary outcomes included major adverse cardiovascular and cerebrovascular events (MACCE), cardiotoxicity events and consequent cancer therapy modifications.

**Results:**

Sixty-eight patients with ACHD and a history of cancer were included in the study. 82% of patients had moderate or great ACHD anatomic complexity. Over a median follow-up of 5 years after cancer diagnosis, 16 (24%) patients died, with 69% of deaths being due to cancer. Univariate predictors of mortality were baseline arrhythmia (OR 3.82, 95% CI 1.15-12.67, *p* = 0.028), baseline diuretic therapy (OR 3.54, 95% CI 1.04-12.04, *p* = 0.044) and advanced cancer stage at diagnosis (OR 2.37, 95% CI 1.32-4.25, *p* = 0.004). MACCE occurred in 40 (59%) patients and was independently predicted by baseline diuretic requirement (OR 9.91, 95% CI 1.12-87.85, *p* = 0.039). A 14% incidence of cardiotoxicity was seen; 3 patients needed modification and 1 patient needed temporary interruption of cancer therapy for 2 weeks.

**Conclusions:**

Considerable mortality occurred in this cohort of patients with ACHD and cancer; most deaths were cancer-related. A high rate of MACCE was observed, yet rates of obligatory modification of cancer therapy due to cardiotoxicity were low.

**Supplementary Information:**

The online version contains supplementary material available at 10.1186/s40959-022-00144-z.

## Introduction

Adults with congenital heart disease (CHD) represent a heterogeneous and rapidly growing population that now comprises the majority of patients with CHD [1]. Adult congenital heart disease (ACHD) patients are known to be at increased risk of death and hospitalization compared to the general population even after correction of their CHD [2]. Malignancy has recently emerged as the fourth leading cause of mortality and the most common non-cardiac cause of death in these patients [3, 4]. Multiple large population studies have further established a 1.5 to 2-fold increased prevalence and incidence of cancer in ACHD compared to the general population [5–8]. This has led the American Heart Association to highlight malignancy as a major non-cardiac pathology in ACHD in a recent scientific statement [9].

However, data guiding clinical management and risk assessment of cancer in ACHD are lacking. There are no studies describing long-term outcomes of these patients after cancer diagnosis, nor are there data on the safety and tolerability of potentially cardiotoxic systemic cancer treatments in the setting of their often complex pre-existing heart disease. We hence sought to fill this data void with a study investigating long-term cardiovascular and cancer-related outcomes, risk factors of adverse outcome and tolerability of cancer therapies in ACHD patients with malignancy.

## Methods

### Study population and design

We conducted a retrospective single-center cohort study comprising all patients > 18 years of age seen at the Ahmanson/UCLA Adult Congenital Heart Disease Center in Los Angeles, California between January 1990 and December 2021 who had a current or past diagnosis of malignancy. Malignancy was defined by the International Classification of Disease, ninth and tenth editions (ICD-9 and ICD-10) diagnostic codes of C00-D09 (ICD-9) and 140-208.91 (ICD-10), similar to prior studies [6, 8]. Exclusion criteria included non-malignant neoplasms, heart transplantation prior to cancer diagnosis, and lack of CHD. Patent foramen ovale was not considered as CHD.

### Data collection

Clinical data were obtained from our electronic medical record and external facilities when available, and supplemented by correspondence with patients, families and treating physicians when needed. Only patients who had consented to use their records for medical research were included in this study. The study protocol was approved by the Institutional Review Board of the University of California, Los Angeles.

Baseline data were gathered as close to the time of cancer diagnosis as possible, and within 3 months of cancer diagnosis. Anatomic complexity was classified as per the ACHD anatomic and physiological classification system formulated by the 2018 ACHD guidelines [10]. Other data obtained included any cardiac surgeries and fluoroscopic procedures, pre-existing arrhythmias, ventricular and valvular function by echocardiography and/or magnetic resonance imaging (MRI) if available, cardiac biomarker levels, New York Heart Association (NYHA) functional class and cardiac medications being taken at the time of cancer diagnosis. Comorbid conditions such as diabetes mellitus, hypertension and liver disease were documented.

Cancer-related data obtained included age, primary location and stage at diagnosis and details of cancer therapy, including doses of known cardiotoxic therapies if available.

Follow-up metrics included vital status, NYHA functional class, hospitalizations since cancer diagnosis, cardiac biomarker data, as well as echocardiography and/or MRI data obtained both at most recent follow-up and at the time of peak B-type natriuretic peptide (BNP) level following cancer diagnosis.

### Outcomes

The primary outcome was all-cause mortality. The key secondary outcome was the occurrence of major adverse cardiovascular and cerebrovascular events (MACCE) at any time after cancer diagnosis, defined as a composite of cardiovascular death, decompensated heart failure, clinically significant arrhythmia requiring pharmacologic or procedural intervention, acute coronary syndrome, spontaneous venous thrombosis/ thromboembolism and stroke. Cancer-related outcomes included malignancy progression, remission and cancer-related mortality.

Cardiotoxicity was defined by the occurrence of therapy-related MACCE, which was determined as such if it was deemed to have a strong association with, or was highly likely to have been caused by cancer therapy. All MACCE were individually reviewed, and determination of causation by cancer therapy was either documented by the treating provider at the time, or independently adjudicated by a cardio-oncologist (EHY). Management of cardiotoxicity and any consequent modifications or interruption to cancer therapy were recorded.

### Statistical analysis

Baseline and outcomes data were reported as frequencies with percentages and medians with interquartile ranges (IQR) when appropriate. Frequencies of pertinent cardiovascular and oncologic covariates in those with and without key outcome events were compared using the Pearson χ2 or Fisher’s exact test when appropriate. These covariates were also assessed as predictors for the primary and secondary endpoints using univariate binary logistic regression via maximal likelihood estimation. When complete separation was encountered, binary logistic regression was performed using the penalized likelihood method described by Firth [11]. Covariates found to predict outcome events were entered into correlation matrices assess correlation, with a correlation coefficient > 0.5 signifying significant correlation. Covariates without significant correlation were then entered into fixed multivariate binary logistic regression models to evaluate for independent predictors of outcome, if/when justified by the number of outcome events. Finally, survival analysis was performed for the primary and secondary outcomes using the Kaplan-Meier method, with the log-rank test used to assess for difference in survival probability of subgroups. A *p*-value of < 0.05 was chosen as the definition for statistical significance. Data analysis was performed using Stata/SE 17.0 (StataCorp LLC, College Station, Texas) and SPSS statistics, Version 27 (IBM).

## Results

Sixty-eight patients with ACHD and malignancy met inclusion criteria, out of a total of 6963 patients seen at our center during the study period, thus yielding a cancer prevalence of 0.98% or 980 per 100,000 individuals. Baseline demographics are detailed in Table 1. The median age of the cohort at the time of cancer diagnosis was 43.5 years (IQR 3-11), while age at most recent follow-up was 53.5 years (IQR 40-66). Four (6%) patients were under the age of 18 years at the time of cancer diagnosis. 59% of the cohort was female, while Caucasian (62%) and Hispanic (18%) races were the most represented. Hypertension and chronic liver disease was noted in 27 and 24% of patients, respectively, with Fontan-associated liver disease, found in 11 patients, being the most common liver pathology. Median follow-up after cancer diagnosis was 5 years (IQR 3-11, range 0.2-51).

**Table 1:** Key baseline cardiovascular data

Characteristic	Frequency (***n*** = 68)
Median age at most recent follow-up, yr (IQR)	53.5 (40-66)
Median age at first cancer diagnosis, yr (IQR)	43.5 (31-60)
Median follow-up since cancer diagnosis, yr (IQR)	5 (3-11)
Female sex (%)	40 (59)
ACHD Anatomic Complexity (%)	
Class I	12 (18)
Class II	32 (47)
Class III	24 (35)
Single ventricle circulation (%)	15 (22)
Fontan palliation (%)	12 (18)
Systemic right ventricle in biventricular circulation (%)	4 (6)
Baseline cyanosis (%)	6 (9)
Prior cardiac surgery (%)	53 (78)
Arrhythmia prior to cancer diagnosis (%)	30 (44)
Supraventricular tachycardia	19 (28)
Atrial fibrillation	12 (18)
Ventricular arrhythmia	1 (2)
Sinus node dysfunction	2 (3)
Atrioventricular nodal block	3 (4)
Heart failure prior to cancer diagnosis (%)	17 (25)
NYHA Functional Class at baseline (%)	
Class I	53 (78)
Class II	13 (19)
Class III	2 (3)
Medications prior to cancer diagnosis, *n* = 66 (%)	
ACE-inhibitor/ angiotensin receptor blocker	16 (24)
Beta-blocker	24 (36)
Diuretic	16 (24)
Clinical Comorbidities	
Diabetes Mellitus	5 (7)
Hypertension	18 (27)
Chronic kidney disease	7 (10)
Coronary artery disease (> 50% luminal stenosis)	6 (9)
Smoking History	13 (19)
Chronic liver disease	16 (24)

*ACE* Angiotensin converting enzyme, *ACHD* Adult congenital heart disease, *IQR* Interquartile range, *NYHA* New York Heart Association

### Baseline cardiovascular data

**Fig. 1:**
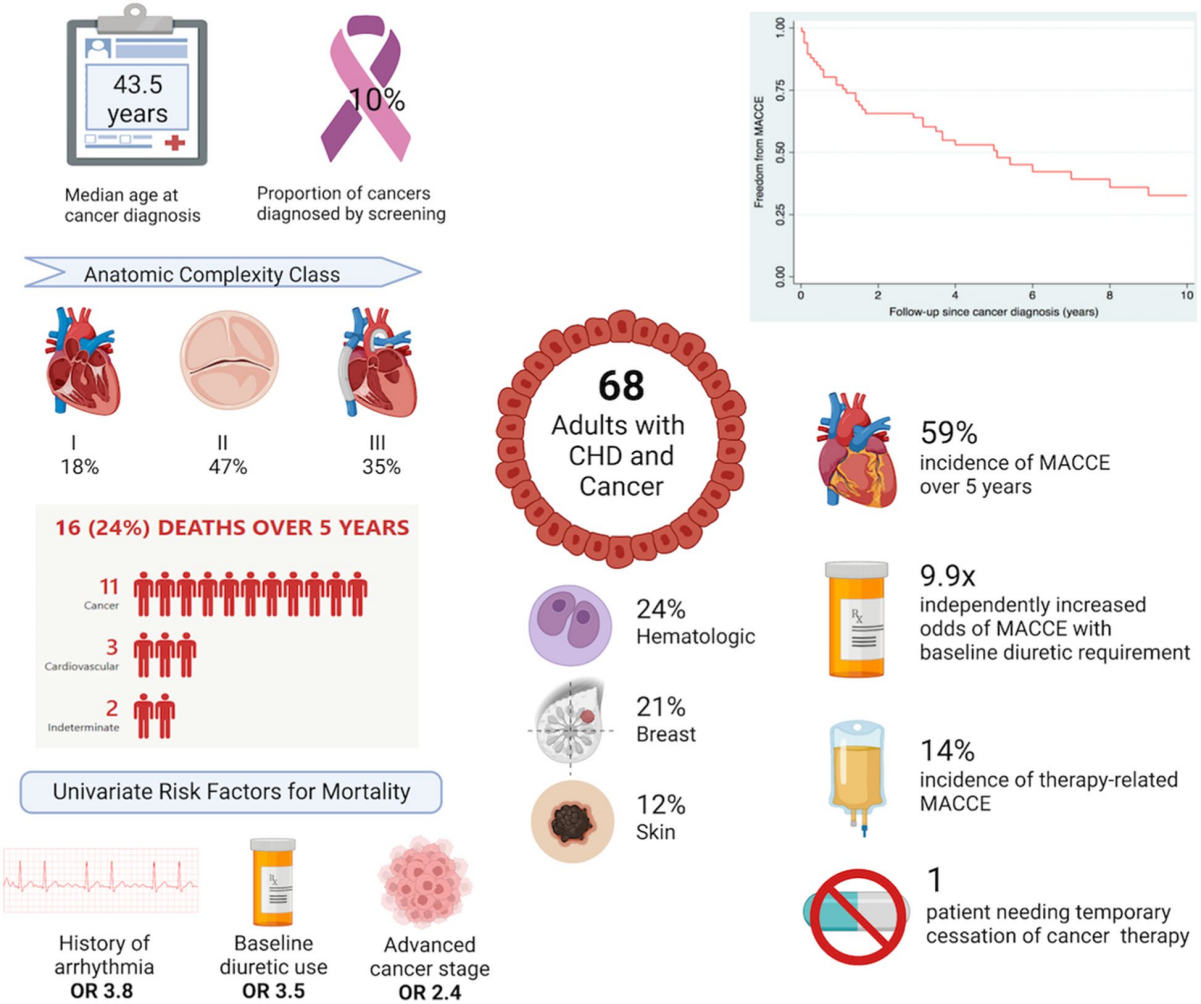
In this young cohort of adults with congenital heart disease and cancer, only a minority of cancers were detected by screening. Despite predominantly moderate or great anatomic complexity, most deaths occurred to cardiovascular causes. Baseline arrhythmia, diuretic requirement and advanced cancer predicted mortality. A high incidence of MACCE was observed, with baseline diuretic therapy being the only independent risk factor. Rates of therapy-related MACCE and consequent cessation of cancer therapy were low. Created with biorender.com

Patients predominantly had moderate or great anatomic complexity of CHD, with 12 (18%) having class I (simple) complexity and 24 (35%) having class III (great) complexity (Fig. 1). Fifteen (22%) patients had single ventricle circulation and 12 (18%) had undergone a Fontan palliation. Prior to cancer diagnosis, 53 (78%) patients had cardiac surgery and 35 (52%) patients had fluoroscopic procedures. History of arrhythmia at the time of cancer diagnosis was present in 30 (44%) patients, with supraventricular tachycardia (28%) and atrial fibrillation (18%) being the most common arrhythmias. Median baseline BNP level was 68 pg/mL (IQR 37-174), while baseline NYHA functional class of I, II and III were noted in 78, 19 and 3% patients respectively (Table 1).

Baseline echocardiographic data were available in 59 patients (Supplemental Table 1). Systemic and subpulmonary ventricular dysfunction were noted in 14 and 15% respectively. Diastolic function was not consistently reported, however, one patient had severe biventricular restrictive cardiomyopathy – all others marked as having ventricular dysfunction had systolic dysfunction. Moderate or greater valvular stenosis/ regurgitation was noted in 53% of echocardiograms, with 23% demonstrating severe valvular lesions. No MRI was performed within 3 months of cancer diagnosis.

### Baseline cancer-related data

**Fig. 2:**
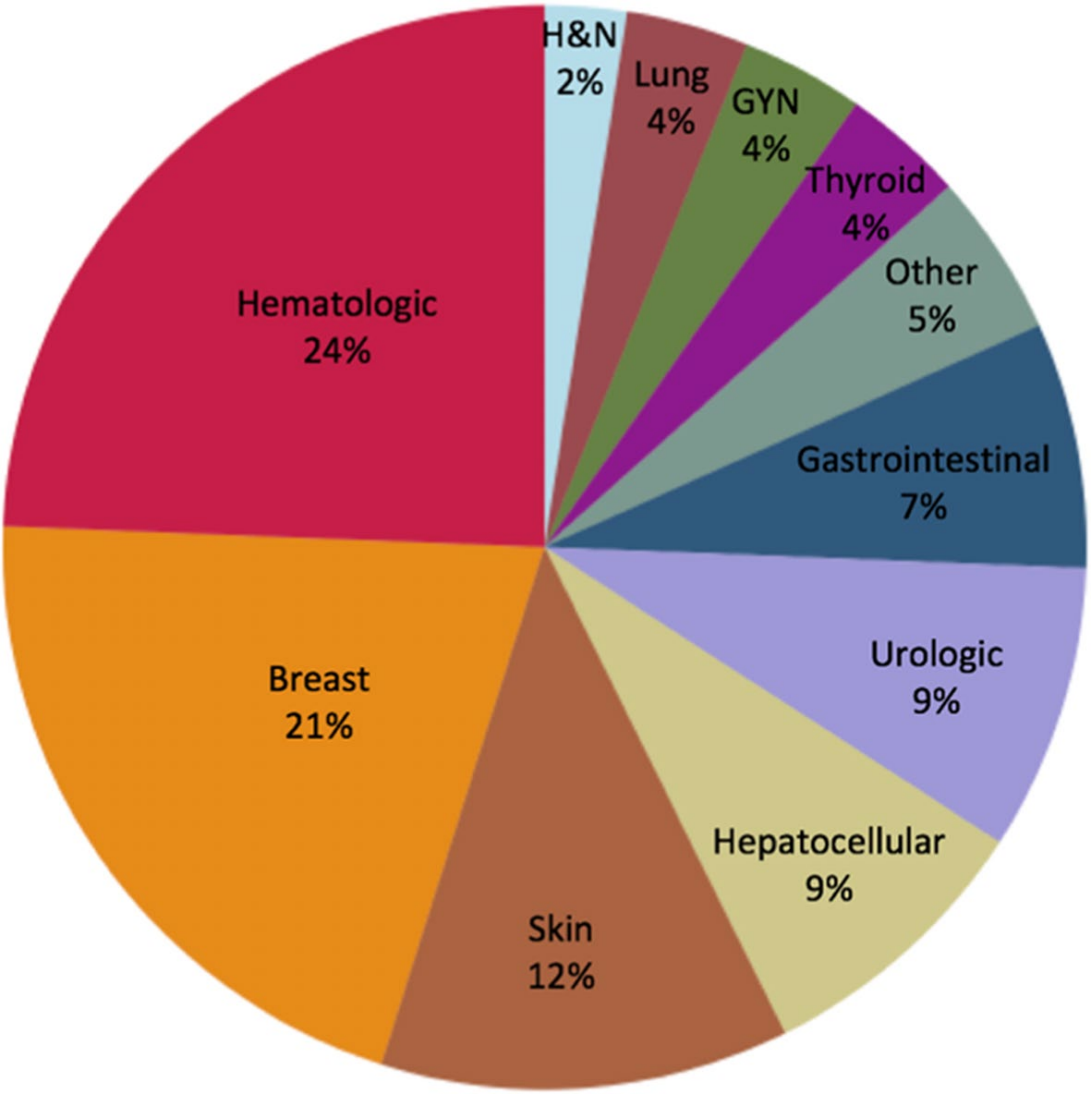
Malignancies in the cohort classified by primary site (*n* = 82). GYN = gynecologic, H&N = head and neck

Eighty-two cancers were diagnosed in 68 patients, with 12 (18%) patients having been diagnosed with multiple cancers (Table 2). Hematologic malignancy was the most common primary cancer accounting for 24% of all cancers, followed by breast (21%) and skin (12%) cancer (Fig. 2). Hepatocellular carcinoma (HCC) was the fourth most common cancer (9%) along with urologic malignancies; 5 out of the 7 patients with HCC had undergone a prior Fontan palliation.

**Table 2:** Cancer-related baseline characteristics and treatment data

Characteristic	Frequency, ***n*** = 68 (%)
Stage at initial cancer diagnosis	
I	23 (38)
II	14 (23)
III	10 (17)
IV	13 (22)
Multiple cancer diagnoses	12 (18)
Genetic abnormalities	
Trisomy 21	4 (6)
Noonan syndrome	1 (1)
BRCA mutation	2 (3)
JAK2 mutation	1 (1)
Any cancer treatment	64 (94)
Surgical resection alone	14 (21)
Radiation therapy	26 (38)
Mediastinal radiation	15 (22)
Systemic pharmacotherapy	41 (60)
Radiofrequency ablation	2 (3)
Taxane/Vinca Alkaloids	16 (24)
Platinum based	12 (18)
Anthracycline	11 (16)
Immune checkpoint inhibitors	7 (10)
HER2 inhibitor	5 (7)
Tyrosine kinase inhibitor	5 (7)
VEGF inhibitor	5 (7)
EGFR inhibitor	2 (3)
BRAF/MEK inhibitor	2 (3)

*VEGF* Vascular endothelial growth factor, *EGFR* Epidermal growth factor receptor

Only 16% of the cohort had undergone any prior cancer screening, and cancer was diagnosed by screening in only 10% of all cases. 38% patients had stage I disease while 22% had stage IV disease at the time of diagnosis. Known genetic mutations prior to cancer diagnosis were present in 7 patients, including 4 with trisomy 21, of whom 3 developed leukemia.

Multiple cancers were diagnosed in 12 (18%) patients; lymphoma was the most common primary malignancy while the most common subsequent malignancies were of breast and skin origin. Two patients were deemed to have had secondary cancers due to treatment administered for their primary cancer (Supplemental Table 2).

### Mortality

Sixteen patients died over the course of follow-up, conferring a 24% all-cause mortality. Eleven deaths (69%) occurred due to non-cardiac causes, all of which were cancer-related (Table 3). Cardiovascular death occurred in 3 patients while 2 patients died of indeterminate causes. Details regarding mortality of specific patients can be found in the Supplemental Appendix.

**Table 3:** Outcome measures of the cohort (*n* = 68 unless otherwise specified)

Outcome measure	Frequency, ***n*** = 68 (%)
Mortality	16 (24)
Cardiac	3/16 (19)
Non-Cardiac	11/16 (69)
Indeterminate	2/16 (12)
MACCE^a^	40 (59)
Arrhythmia	29 (43)
Decompensated heart failure	22 (32)
Thromboembolism (arterial or venous)	7 (10)
Stroke	3 (4)
Hospitalization due to MACCE	34 (50)
Worsening systemic ventricular function	9/61 (15)
Worsening subpulmonary ventricular function	8/53 (15)
Worsening valvular function	25/62 (40)
Worsening BNP	28/33 (85)
Worsening NYHA Functional Class	19 (28)
Cardiac transplantation^b^	3 (4)
Cancer Outcome (index malignancy)	
Progression	17 (25)
Relapse	3 (4)
Partial Remission	26 (38)
Complete Remission	12 (18)
Stable and undergoing treatment	10 (15)

*MACCE* Major Adverse Cardiovascular and Cerebrovascular Event, *BNP* B-type Natriuretic Peptide, *NYHA* New York Heart Association

^a^More than one MACCE event may have occurred in the same patient

^b^One patient underwent combined heart and liver transplantation

**Fig. 3:**
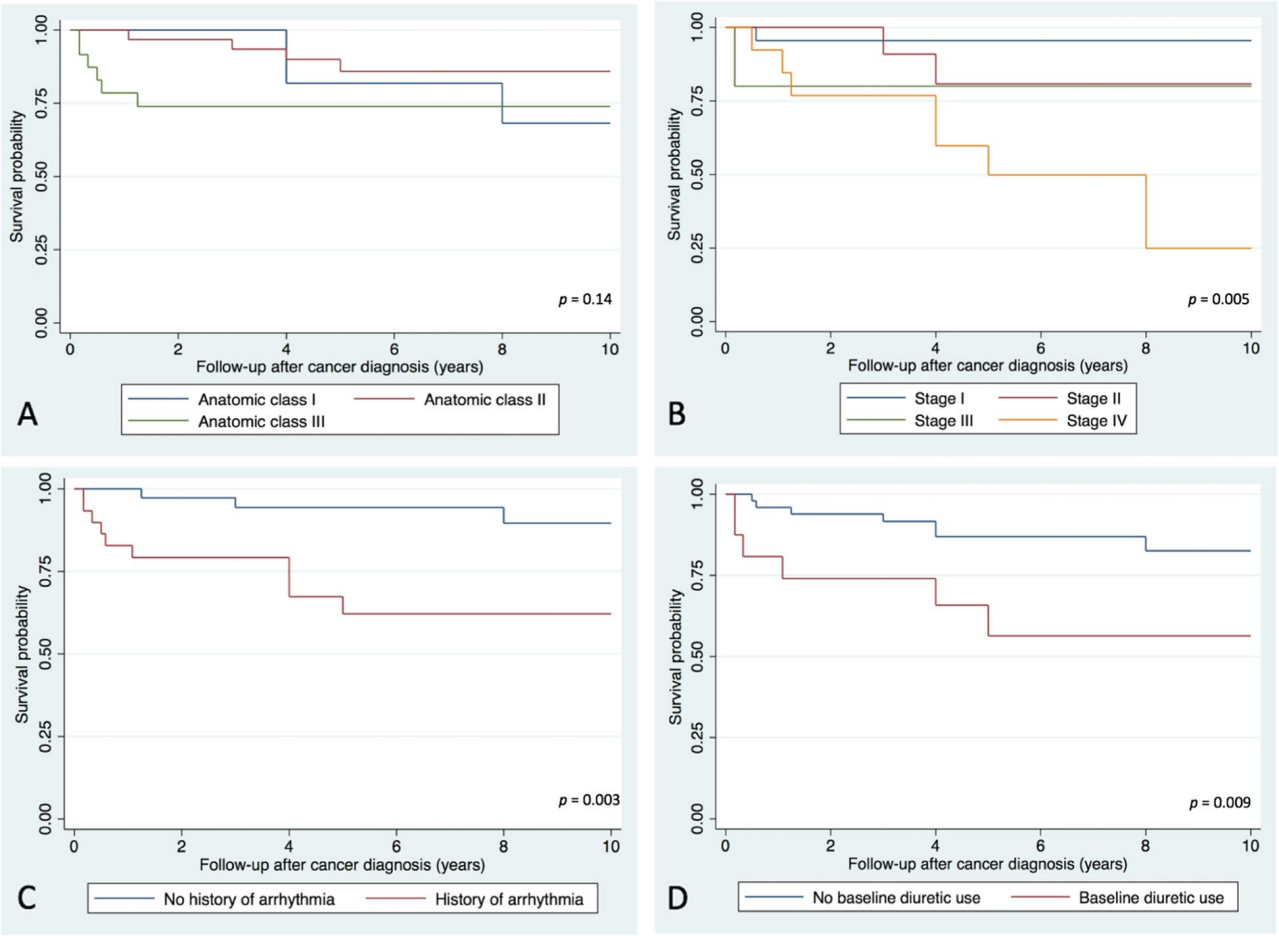
Kaplan-Meier survival curves evaluating survival probability 10 years after cancer diagnosis, stratified by ACHD anatomic class (**A**), stage of cancer at diagnosis (**B**), history of arrhythmia (**C**) and baseline diuretic use (**D**)

Survival probability at 1, 5, and 10 years was 93, 82 and 77% respectively. Univariate predictors of mortality were incrementally advanced cancer stage at diagnosis (OR 2.37, 95% CI 1.32 – 4.25, *p* = 0.004), history of arrhythmia prior to cancer diagnosis (OR 3.82, 95% CI 1.15 - 12.67, *p* = 0.028) and diuretic use at baseline (OR 3.54, 95% CI 1.04 - 12.04, *p* = 0.044) (Table 4)*.* Immune checkpoint inhibitor (ICI) therapy use also significantly increased odds of mortality (OR 11.36, 95% CI 1.95 - 66.38, *p* = 0.004), however all patients with ICI use had cancer stage of stage II or greater at the time of administration and ICI use correlated with metastatic disease at the time of diagnosis (*r* = 0.34). Survival analysis showed significantly reduced survival in the subgroups with advanced cancer i.e. stage IV compared to stages I-III (*p* = 0.005), history of arrhythmia (*p* = 0.003) and baseline diuretic use (*p* = 0.009), while there was no significant difference in survival stratified by ACHD anatomic complexity (*p* = 0.14) (Fig. 3).

**Table 4:** Univariate predictors of mortality and MACCE; rows in bold denote significance (*p* < 0.05)

Predictor variable	Mortality (***n*** = 16)		MACCE (***n*** = 40)	
	OR (95% CI)	*p*	OR (95% CI)	*p*
Male sex	0.87 (0.28 - 2.70)	0.811	1.03 (0.38 - 2.79)	0.952
ACHD complexity	1.22 (0.54 - 2.72)	0.632	1.52 (0.75 - 3.09)	0.246
Single ventricle circulation	1.24 (0.33 - 4.62)	0.748	**6.02 (1.23 - 29.37)**	**0.011**
Prior cardiac surgery	5.53 (0.67 - 45.81)	0.054	1.985 (0.62 - 6.34)	0.246
Number of fluoroscopic procedures	1.02 (0.74 - 1.40)	0.928	**1.46 (1.04 - 2.06)**	**0.018**
History of arrhythmia	**3.82 (1.15 - 12.67)**	**0.028**	**2.90 (1.02 - 8.17)**	**0.039**
Systemic ventricular dysfunction at baseline	2.5 (0.49 - 12.76)	0.271	13.33 (0.73 - 247.15)^a^	0.081
Subpulmonary ventricular dysfunction at baseline	0.56 (0.06 - 5.37)	0.601	6.63 (0.73 - 60.22)	0.093
Valvular dysfunction (> mild)	1.25 (0.38 - 4.11)	0.713	1.21 (0.42 - 3.47)	0.724
NYHA class at baseline	1.81 (0.65 - 5.09)	0.259	2.25 (0.71 - 7.09)	0.168
ACE-I/ARB use at baseline	1.06 (0.29 - 3.89)	0.935	1.15 (0.360 - 3.67)	0.814
Diuretic use at baseline	**3.54 (1.04 - 12.04)**	**0.044**	**15.62 (1.91 - 127.64)**	**0.001**
Beta blocker use at baseline	1.07 (0.33 - 3.42)	0.914	2.87 (0.94 - 8.66)	0.055
Multiple Cancer diagnoses	0.68 (0.13 - 3.54)	0.640	2.0 (0.48 - 8.35)	0.326
Cancer stage at diagnosis	**2.37 (1.32 - 4.25)**	**0.004**	0.80 (0.51 - 1.25)	0.325
Any systemic cancer therapy use	2.38 (0.68 - 8.36)	0.160	1.03 (0.38 - 2.79)	0.952
Platinum based compounds	0.59 (0.11 - 3.00)	0.521	0.89 (0.25 - 3.18)	0.859
Kinase inhibitor	2.44 (0.67 - 8.95)	0.178	1.05. (0.30 - 3.65)	0.939
Immune Checkpoint Inhibitor use	**11.36 (1.95 - 66.38)**	**0.004**	1.39 (0.24 - 8.17)	0.713
Anthracycline use	1.27 (0.29 - 5.49)	0.753	0.63 (0.16 - 2.42)	0.501
Mediastinal radiation therapy	0.77 (0.19 - 3.16)	0.711	1.47 (0.44 - 4.90)	0.529

*OR* Odds ratio, *CI* Confidence interval. *ACE-I* Angiotensin converting enzyme inhibitor, *ARB* Angiotensin receptor blocker, *MACCE* Major Adverse Cardiovascular and Cerebrovascular Event, *NYHA* New York Heart Association

^a^Firth logistic regression used due to complete separation

### MACCE

Over the follow-up period, MACCE occurred in 40 (60%) patients and prompted hospitalization in 34 (50%). The events were driven mostly by arrhythmias, which occurred in 29 (43%) patients, and decompensated heart failure which occurred in 22 (32%) patients (Table 3). Thromboembolic events occurred in 7 (10%) patients. The most common arrhythmias were atrial fibrillation and supraventricular tachycardia seen in 23 and 19% of the patients respectively. Twenty of the 29 patients with arrhythmia events did not have a baseline history of arrhythmia. Of the 22 patients who developed decompensated heart failure, 9 did not have a history of heart failure prior to cancer diagnosis. Heart failure hospitalization occurred in 17 (25%) patients. Two patients underwent heart transplantation, while a third underwent combined heart and liver transplantation and died post-operatively.

**Fig. 4:**
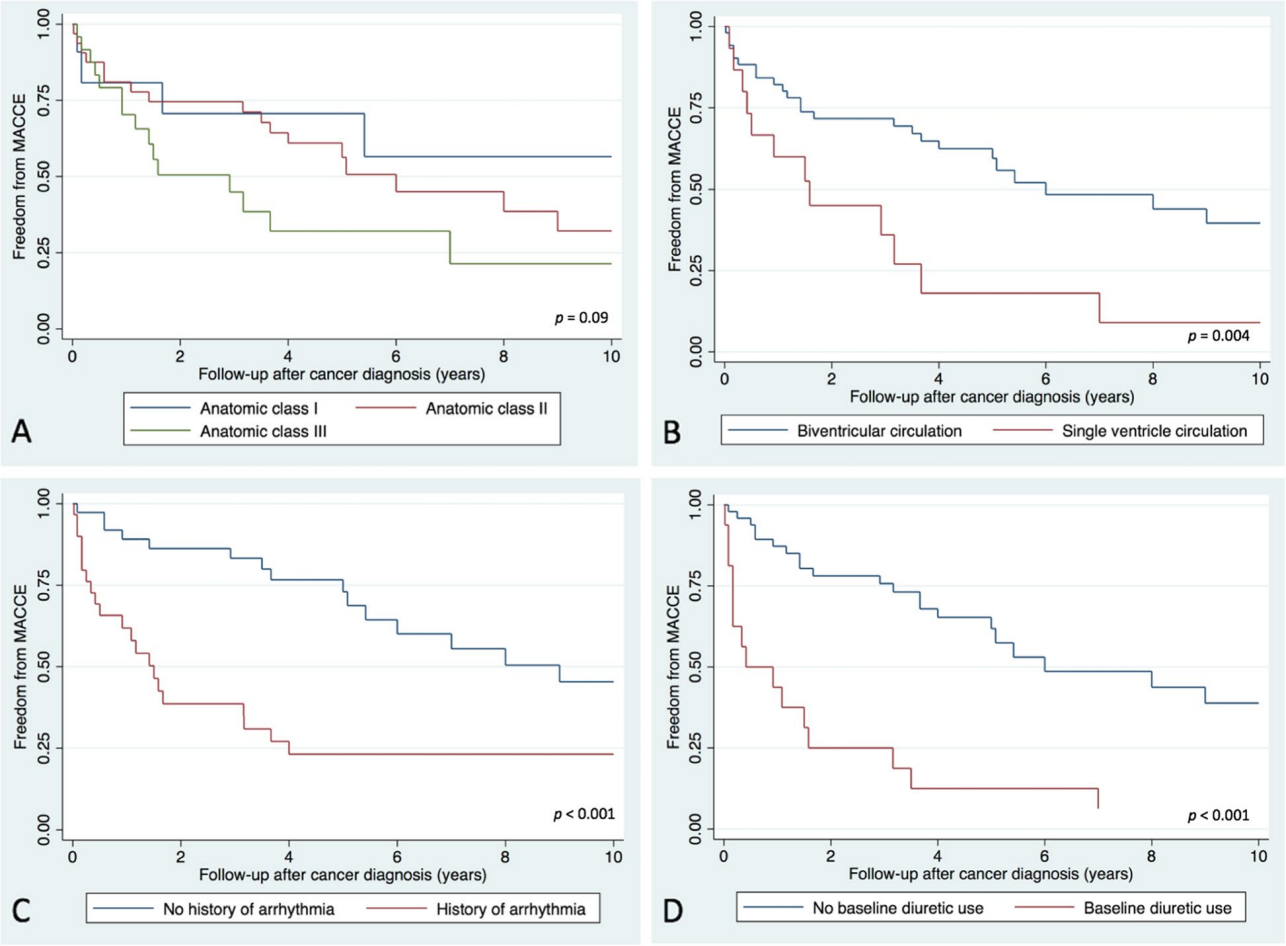
Kaplan-Meier survival curves evaluating freedom from MACCE 10 years after cancer diagnosis, stratified by ACHD anatomic class (**A**), single versus biventricular circulation (**B**), history of arrhythmia (**C**) and baseline diuretic use (**D**)

Freedom from MACCE at 1, 5 and 10 years after cancer diagnosis was 77, 53 and 33% respectively (Fig. 1). Univariate predictors of MACCE were baseline diuretic use (OR 15.62, 95% CI 1.91 - 127.64, *p* = 0.001), single ventricle circulation (OR 6.02, 95% CI 1.23 - 29.37, *p* = 0.011), history of arrhythmia (OR 2.90, 95% CI 1.03 - 8.17, *p* = 0.039) and number of fluoroscopic procedures (OR 1.46, 95% CI 1.04 - 2.06, *p* = 0.018) (Table 4). Number of fluoroscopic procedures correlated with single ventricle circulation (*r* = 0.53) and baseline arrhythmia (*r* = 0.50). In multivariable analysis, baseline diuretic use was the only independent predictor of MACCE (OR 9.91, 95% CI 1.12 - 87.85, *p* = 0.039) (Table 5)*.* A significantly reduced freedom from MACCE was seen in subgroups with single ventricle anatomy (*p* = 0.004), history of arrhythmia prior to cancer diagnosis (*p* < 0.001) and baseline diuretic use (*p* < 0.001). Ten-year freedom from MACCE was lower with class II and class III anatomic complexity, however this was not statistically significant (*p* = 0.09) (Fig. 4).

**Table 5:** Multivariable predictors of MACCE

Variable	Odds Ratio	95% Confidence interval		Standard Error	*p*
		Upper limit	Lower limit		
History of arrhythmia	1.54	0.48	4.96	0.92	0.466
Baseline diuretic use	**9.91**	**1.12**	**87.85**	11.03	**0.039**
Single ventricle anatomy	2.73	0.48	15.51	2.42	0.258

*MACCE* Major Adverse Cardiovascular and Cerebrovascular Events

### Echocardiography

At most recent follow-up, systemic ventricular dysfunction was noted in 10/67 (15%) patients, while 11/57 (19%) patients with biventricular circulation had subpulmonary ventricular dysfunction. When compared to baseline, systemic ventricular dysfunction was worse in 9/10 patients and subpulmonary ventricular dysfunction worse in 8/8 patients with baseline data. Moderate or greater valve dysfunction was present in 38/67 (57%) patients at most recent follow-up (Table 3).

### Cancer therapeutics and outcomes

Details of cancer therapy are presented in Table 2. Malignancy was treated in 64 patients; the remaining 4 patients either were managed conservatively or died before receiving treatment. Fourteen patients with locally advanced malignancy received surgical resection as their sole cancer treatment, with all achieving complete remission without additional treatment (Table 3). Systemic pharmacologic therapy was administered to 41 (60%) patients, with alkylating and alkylating-like agents being the most common (42% patients), followed by antimetabolites (30%) and taxanes/ vinca alkaloids (30%). Median cumulative dose of anthracyclines (doxorubicin equivalent) in 8 patients with available data was 179 mg/m2. Mediastinal radiation was administered to 15 (22%) patients. Stem cell transplantation was performed in 5 (7%) patients. No patient received chimeric antigen T-cell therapy.

**Fig. 5:**
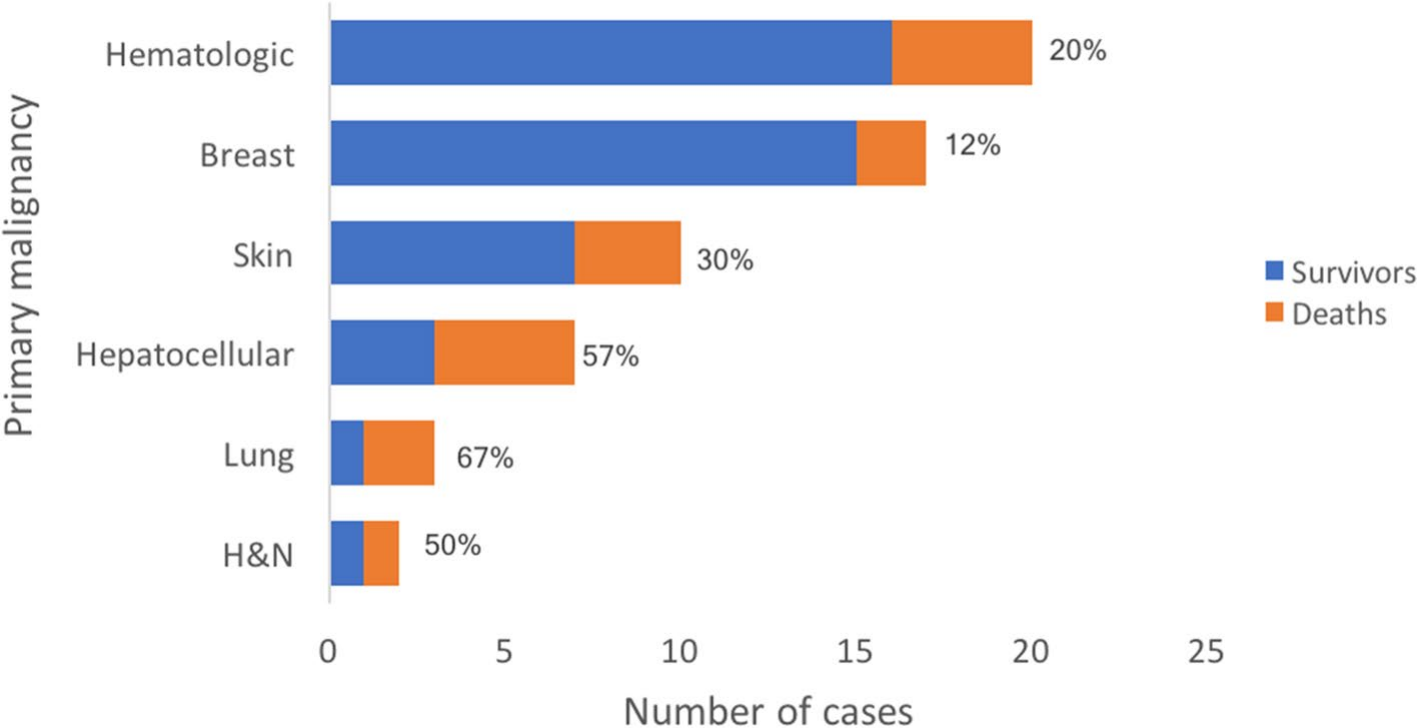
Mortality in the cohort stratified by primary malignancy, along with case fatality rates for each malignancy. More recent malignancy was chosen for patients with multiple cancers. Non-fatal malignancy sub-types are not depicted. H&N = head and neck

Over the follow-up period, 17 (25%) patients had cancer progression, 26 (28%) patients achieved partial remission and 12 (18%) achieved complete remission. Cancer relapse occurred in 3 (4%) patients while 10 (15%) patients had stable disease with either ongoing or maintenance therapy at the time of most recent follow-up (Table 3). When stratified by primary malignancy, case fatality was highest in patients with lung cancer, with 2 deaths in the 3 diagnosed patients (Fig. 5). HCC had the second highest fatality rate of 57%, while breast cancer carried the least case fatality of all fatal malignancies, with 2/17 (12%) deaths.

### Cardiotoxicity

A total of 7 therapy-related MACCE occurred in 6 patients, i.e. 14% of patients on systemic cancer therapy, and 9% of the entire cohort. Out of the 7 events, 5 were decompensated heart failure, with one patient experiencing 2 episodes in response to different therapeutic agents. Ventricular function deteriorated in only 1 of these 5 events, in a patient with possible ICI-associated myocarditis. Three of these events occurred in patients with pre-existing cardiomyopathy and/or valvulopathy. In response to the MACCE event, cancer therapy had to be modified in 2 patients and completely withheld in one patient for 2 weeks until treatment of the heart failure episode. In most patients, therefore, continuation of the current therapy was made possible. Additional details of the presentation and management of therapy-associated MACCE are found in Table 6 and the supplementary appendix.

**Table 6:** Cancer therapy-related MACCE events in the cohort, adjudicated as cardiotoxicity events

Patient ID	ACHD diagnoses	MACCE prior to cancer therapy	Primary cancer	Cancer Therapy	Therapy-related MACCE	Time of therapy-related MACCE episode from initiation of therapy	Management of suspected cardiotoxicity	Outcome of cardiotoxic episode	Cancer-related outcome
3	• ASD • VSD • Subaortic membrane	None	Melanoma	Ipilimumab/ nivolumab	New non-ischemic cardiomyopathy with heart failure. LVEF decreased from 53 to 36% after first cycle of immunotherapy	31 days	• Ipilimumab discontinued, switched to trametinib. • Furosemide, carvedilol, spironolactone and lisinopril started	• Improvement in LV EF and volume status. • Able to tolerate additional cycles of therapy	• New brain metastases discovered • Suffered large intracranial hemorrhage due to gamma-knife treatment • Died shortly after comfort care initiated
11	• Bicuspid aortic valve • Mitral valve prolapse	• SVT, paroxysmal AF • Known LV cardiomyopathy (LVEF 35%) due to ventricular pacing/ mid-LAD 70% plaque	Mantle cell lymphoma	Bendamustine/ rituximab (BR)	Decompensated heart failure, with 1.2 L administration of fluids with first cycle	Same day as initiation	Prophylactic furosemide before each subsequent cycle	• Fluid status well-managed, no further decompensation or change in LV function. • Completed 4 cycles of BR.	Remission followed by relapse
11	• Bicuspid aortic valve • Mitral valve prolapse	Same as above	Mantle cell lymphoma	Ibrutinib	Decompensated heart failure. No changes to LV EF or valve function	30 days	• Discontinuation of ibrutinib, • Initiation of bendamustine/ rituximab (BR) with scheduled hospitalization for management of fluid status with each dose	• Fluid status improved. • Subsequent doses of BR given with intravenous furosemide during scheduled admissions • Tolerated BR therapy well	• Progression to blast crisis • Died shortly after comfort care initiated
21	Bicuspid aortic valve	None	Hodgkin’s lymphoma	Mediastinal radiation	• Radiation pericarditis • Radiation induced severe valvulitis of the aortic, tricuspid and mitral valves	Unknown	• Pericardial window • surgical tricuspid valve replacement.	• Worsening heart failure due to severe mitral and aortic regurgitation. • Deemed unintervenable due to poor functional status from metastatic lung cancer	• Hodgkin’s lymphoma with remission • Metastatic small-cell lung cancer diagnosed approximately 35 years later, progressed despite immunotherapy. • Died shortly after being placed on palliative care for advanced cancer and heart failure
41	• Double outlet right ventricle (unintervened) • Pulmonic stenosis • VSD	Paroxysmal AF	Urothelial carcinoma	Gemcitabine/ cisplatin	• Acute chest pain with elevated troponin-I level to 0.86 ng/mL in setting of gastrocnemius vein thrombosis. • No pulmonary embolism found	28 days	Conservative management advised since symptoms resolved.	• None, no recurrence in symptoms and hence no additional ischemia evaluation pursued • Received another 2 cycles of gemcitabine/ cisplatin	• Completed 3 cycles of neoadjuvant gemcitabine/ cisplatin • Underwent robotic nephroureterectomy and cystectomy. Currently under surveillance
44	Bicuspid aortic valve	• None • Known severe aortic regurgitation with LV dilation and preserved LVEF	B-cell acute lymphoblastic leukemia	• Stem cell transplantation, thiotepa and fludarabine. • Previously received ABFM induction including 18.75 mg/m2 of anthracycline + 2 cycles of blinatumomab.	Acute pulmonary edema resulting in hypoxic respiratory failure	10 days after stem cell transplantation, 6 months after induction	Aggressive high-dose intravenous diuretic therapy instituted	• Normalization of volume status • Maintenance oral diuretic therapy instituted • Continued on losartan and carvedilol	• Relapsed 6 months after stem cell transplantation • Currently back on blinatumomab therapy
58	Pulmonary valve stenosis	• None. • Known severe pulmonary regurgitation with normal right ventricular systolic function	Concomitant invasive ductal and lobular carcinoma in the same breast	TCH (taxotere/ carboplatin, herceptin)	• Progressive right-sided heart failure with each cycle, worst after completion of 6th and final cycle of TCH. • No changes to ventricular systolic function on echocardiogram	21 days after 1st cycle	Oral diuretic therapy	• Normalization of volume status and resolution of heart failure • Maintenance diuretic therapy instituted	• Underwent prophylactic right breast mastectomy. • Currently in remission

*ASD* Atrial septal defect, *VSD* Ventricular septal defect, *SVT* Supraventricular tachycardia, *AF* Atrial fibrillation, *LAD* Left anterior descending, *LV* Left ventricle, *EF* Ejection fraction

Of the 42 patients who received systemic cancer therapy, 2 patients were on cardioprotective medications at baseline while 3 others were pre-emptively started on a cardioprotective medication regimen (angiotensin-converting enzyme inhibitor and beta blocker) before chemotherapy. None of these patients experienced therapy-related MACCE. Additionally, none of the patients receiving anthracyclines, and one of the patients on anti-HER2 therapy had therapy-related MACCE.

## Discussion

This is, to our knowledge, the first study to evaluate long-term outcomes, delineate predictors of adverse outcomes and describe tolerability of cancer therapies in ACHD patients with malignancy. Our study yields several important findings that will be of value to cardiologists as well as oncologists caring for this complex patient population.

### Cancer risk

Ours was a high-risk patient cohort, from both standpoints of malignancy and cardiovascular disease. Even though 94% of the patients had cancer diagnosed as adults, the median age at diagnosis was only 43.5 years – much younger than the median age at diagnosis in the United States of 66 years [12]. The prevalence of cancer in our ACHD center was 980 per 100,000 persons – nearly four times that of the national rate of approximately 250 per 100,000 persons for the age group of 40-45 years at diagnosis [12]. Furthermore, 18% of the patients had more than 1 malignancy during the follow-up period, indicating that the issue of cancer risk in ACHD does not end at diagnosis of the first cancer. This is likely due to carcinogenic effects of therapy of the index malignancy, as well as potential cumulative effects of low-dose ionizing radiation (LDIR) exposure from serial imaging and procedures, which often continues throughout adult life in these patients and has been demonstrated to incrementally increase cancer risk in a large population study [13]. LDIR is known to increase risk of multiple cancer types including hematologic, skin and breast – the three most common primary cancers seen in our cohort [13–15]. The fourth most common cancer in our cohort was HCC, which was mostly seen in Fontan patients and carried the second-highest fatality rate. The poor prognosis of this known sequela of Fontan physiology has been well documented, and awareness of the same is critical for all caring for this special population [16, 17]. Furthermore, the recently demonstrated survival benefit of combination regimens consisting of ICI and vascular endothelial growth factor receptor inhibition in advanced HCC holds special promise for Fontan patients, and must be explored further in the context of efficacy and cardiotoxicity [18].

Our study also highlights the concerning deficiency of guideline based cancer screening among ACHD patients. At the time of diagnosis, only 16% of our patients had received prior cancer screening, only 10% cancers were diagnosed by screening and 22% had stage IV disease at the time of diagnosis. These data point not only to the importance of routine primary care follow-up, but also to the broader issue of the lack of established screening protocols to address the already well-established heightened risk of cancer in these patients. The younger age at cancer diagnosis in our cohort compared to the general population suggests that screening should start at an earlier age in ACHD patients, yet no specific cancer screening guidelines apart from current breast cancer guidelines advocate for earlier screening in patients with prior radiation exposure [9, 19]. The development of targeted cancer screening guidelines for ACHD at elevated cancer risk must hence be prioritized.

### Mortality

The 24% mortality in our cohort was mostly driven by cancer-related deaths despite the elevated baseline cardiovascular risk – most patients had either moderate or great ACHD complexity, and baseline rates of arrhythmia and heart failure were considerable. These data put into perspective the serious risk that cancer poses to this population, and underscore not only the need for improved diagnosis but also urgent consideration and initiation of treatment in all patients, regardless of CHD complexity. Indeed, ACHD anatomic complexity did not predict mortality; rather, we found that advanced cancer stage at diagnosis more than doubled risk of mortality. ICI use in our cohort was likely a proxy for advanced cancer rather than a causative risk factor for mortality, especially since ICI toxicity (myocarditis) was suspected in only one patient.

Interestingly, history of arrhythmia and baseline diuretic use were also associated with mortality, even though cardiovascular mortality was rare. Baseline diuretic use was required for heart failure in all patients, and heart failure has been recently shown to confer increased risk of cancer-related mortality compared to matched controls [20]. In addition, atrial fibrillation in particular has been shown to increase mortality in breast cancer patients and in those after surgical resection for lung and esophageal cancers [21–23]. It is likely that baseline history of arrhythmia and diuretic requirement were both markers for reduced physiologic reserve that predisposed our patients to cancer-related deaths. Though NYHA functional class did not predict mortality, physiological classification according to the current guideline-recommended ACHD anatomic-physiologic classification system may have been a more wholesome representation of physiologic reserve in our cohort, though this was unable to be assessed due to incomplete baseline data [10].

### MACCE

The 60% incidence of MACCE in our cohort coupled with 50% MACCE-related hospitalization is concerning. To a considerable extent, this is reflective of the underlying complexity and elevated baseline cardiovascular risk of our patients. Incidence of MACCE in adult cohorts of complex CHD is known to be comparable – the incidence of arrhythmia in a cohort of over 900 adults with a 15-year median follow-up after Fontan palliation was 41%, while the 20-year arrhythmia-free survival after atrial switch repair for D-transposition of the great arteries is as low as 40% [24, 25]. The added physiologic stress caused both by malignancy and its treatments also predisposed our patients to exacerbations of arrhythmia or volume overload. Furthermore, we used a broader definition of MACCE compared to other cohorts in the ACHD literature, specifically including outpatient diuretic intensification, which has been repeatedly shown to increase adverse outcome in non-congenital heart failure populations [26, 27]. Our finding of baseline diuretic requirement as a strong, independent predictor of MACCE is clinically meaningful, as this could be used as an easily identifiable qualifier to define increased risk and perhaps institute stricter surveillance in ACHD patients after cancer diagnosis.

Fourteen percent patients on systemic cancer therapy or radiation had therapy-related MACCE. We used this as a broad definition of cardiotoxicity to capture as many events as possible, as opposed to more conventional definitions mandating decrements in ventricular ejection fraction, which would have missed four out of the five heart failure events seen in our study where ejection fraction was unchanged from baseline [28]. Episodes of decompensated heart failure may have been either related to direct toxic effects of cancer therapy on cardiomyocytes or as a consequence of intravenous fluid volume administered during chemotherapy infusions. Our approach to treatment of these patients was proactive attenuation of toxicities and aimed at resuming cancer therapy safely and as soon as possible. All heart failure events resolved with prompt diuresis, and therapy interruption was minimal. We show that cancer therapy including potentially cardiotoxic agents can be tolerated even in complex CHD patients, with frequent outpatient monitoring coupled with close collaboration with oncology and cardio-oncology providers. We advocate for such an approach, given data from a large lung cancer cohort without ACHD showing that patients with concomitant heart failure are significantly less likely to receive any form of treatment, and the fact that most deaths in our own cohort were cancer-related despite high rates of therapy [29].

### Limitations

Our study has several limitations. The sample size limited risk assessment, since factors such as baseline ventricular dysfunction or significant valve disease that had low event rates would otherwise be expected to influence outcome. While our definition of therapy-related MACCE was intentionally broad to capture as many cardiotoxicity events as possible, it was based in part on the temporal relationship between initiation of therapy and the MACCE. Remote cardiotoxic events occurring late after initiation of therapy would have hence been missed. We were unable to assess effects of specific classes of cardiotoxic agents on cardiac function for the same reason. Our approach of using ICD code data inherently assumes universal coding of cancer in the record of all patients with a history of cancer, which may not have been the case especially in patients who had a remote history of successfully treated cancer. Speckled tracking strain imaging, generally regarded as a more sensitive method for monitoring for subclinical cardiotoxicity, was not routinely obtained in our patients, though this technique is yet to be validated in the ACHD population undergoing cancer treatment and is a potential future avenue of study. Finally, our study is limited by the lack of a control group of ACHD patients without cancer, though appropriate matching given the vast heterogeneity within the subtypes and management strategies of CHD is inherently limited, and one can reasonably surmise that a control group without cancer would have significantly less long-term mortality since mortality in this cohort was largely driven by cancer.

## Conclusions

In this cohort of ACHD patients with cancer, cancer diagnosis occurred at a young age, and cancer screening rates were considerably low. Nearly 1 in 4 patients died, with most deaths occurring due to cancer despite significant CHD complexity and baseline cardiovascular risk. We found high rates of incident MACCE after cancer diagnosis, but only a minority of these were considered therapy-related cardiotoxic events. We show that it is feasible to administer systemic cancer therapy to such a cohort and manage cardiotoxicity without prolonged discontinuation of therapy. Future studies of a prospective, multi-center nature are required to assess tolerability of specific therapeutic agents, investigate strategies to better diagnose and mitigate cardiotoxicity and – perhaps most importantly – develop targeted screening protocols for timely diagnosis of cancer in this high-risk population.

## Supplementary Information

**Additional file 1:** Supplementary appendix containing echocardiographic data, escription of invididual patients with non-cancer-related death and multiple cancer diagnoses, and cardiotoxicity management.

## Data Availability

Our original dataset contains confidential PHI but upon request deidentified raw data can be provided.

## Abbreviations

CHD: Congenital Heart Disease
ACHD: Adult Congenital Heart Disease
ICD: International Classification of Disease
MRI: Magnetic Resonance Imaging
NYHA: New York Heart Association
BNP: B-type Natriuretic Peptide
MACCE: Major Adverse Cardiovascular and Cerebrovascular Events
IQR: Interquartile Range
HCC: Hepatocellular Carcinoma
OR: Odds Ratio
CI: Confidence Interval
ICI: Immune Checkpoint Inhibitor
LDIR: Low Dose Ionizing Radiation
