# Supplementary material for: Outcomes of malignancy in adults with congenital heart disease: a single center experience
Source: Cardiooncology. 2022 Nov 23;8:20. doi: 10.1186/s40959-022-00144-z (PMC9685873; doi:10.1186/s40959-022-00144-z)
Supplement: Supplementary file 1 — Additional file 1: Supplementary appendix containing echocardiographic data, escription of invididual patients with non-cancer-related death and multiple cancer diagnoses, and cardiotoxicity management. [file 40959_2022_144_MOESM1_ESM.docx]

Supplementary Appendix

**Subject Page**

Baseline Echocardiographic data ..............................................................................................2

Patients with cardiovascular mortality and mortality

due to indeterminate cause.........................................................................................................3

Valve function at most recent follow-up....................................................................................5

Multiple cancer diagnoses..........................................................................................................7

Cardiotoxicity.............................................................................................................................8

| **Parameter** | **Frequency** |
| --- | --- |
| Systemic ventricular dysfunction (%) | 7/59 (14) |
| Mild | 3 |
| Moderate | 3 |
| Severe | 1 |
| Subpulmonary ventricular dysfunction (%) | 7/48 (15) |
| Mild | 4 |
| Moderate | 2 |
| Severe | 1 |
| Moderate or severe valvular dysfunction (%) | 31/58 (53) |
| Moderate | 19 |
| Severe | 12 |

**Supplemental Table 1:** Baseline echocardiographic data

*Cardiovascular Mortality*

Three patients were adjudged to have had cardiovascular mortality, with cancer having played a significant role in the death of two of these patients:

1. One patient died of heart failure from radiation-induced mitral and aortic regurgitation deemed to be inoperable due to the presence of concomitant stage IV lung cancer.
2. Another patient with failing Fontan circulation, cirrhosis and recurrent ascites developed HCC that caused portal vein thrombosis and accelerated hepatic and cardiac failure.
3. The only patient who did not have a direct cancer-related death was a patient with a well-functioning Fontan circulation who underwent combined orthotopic heart and liver transplantation recommended for HCC. He died from post-operative sepsis that occurred due to prolonged delay in chest closure, owing to right ventricular failure resulting from a stricture at the pulmonary artery anastomotic site.

*Mortality from Indeterminate Causes*

The cause of death in two patients could not be ascertained despite chart review and extensive efforts to obtain additional data from primary and secondary sources. These included:

1. A 19-year-old man with remote surgical closure of ventricular septal defect and no cardiac sequelae who presented to an outside hospital with acute dyspnea and died the same day in the emergency department. He had relapsed Hodgkin’s lymphoma and had received 34 cycles of nivolumab salvage therapy over the preceding 28 months without any known cardiopulmonary complications. Autopsy was not performed and his next of kin was not reachable for permission to obtain medical records
2. A 92-year-old man with stage 0 chronic lymphocytic leukemia and severe bicuspid aortic stenosis status post two bioprosthetic surgical aortic valve replacements who died at home without any available records. Multiple attempts to contact next of kin were unsuccessful.

*Valve function upon follow-up*

Worsening valvular dysfunction occurred in 25/62 (40%) patients with baseline and follow-up data; and moderate or greater valve dysfunction was present in 38/67 (57%) patients at most recent follow-up (table 4). Three patients required transcatheter valve replacements, two in the tricuspid and one in the aortic position. One patient with a remote surgical mitral bioprosthesis developed severe paravalvular regurgitation due to partial dehiscence of the valve ring, and developed worsening systemic left ventricular dysfunction. He underwent successful percutaneous device closure of the paravalvular leak, and achieved recovery of left ventricular systolic function.

| **ID** | **Index malignancy** | **Subsequent malignancies** | **Time interval between malignancy diagnoses (years)** | **Therapy- related malignancy** | **Therapy-related MACCE** | **Mortality** |
| --- | --- | --- | --- | --- | --- | --- |
| 18 | Follicular cancer (thyroid) | Chronic lymphocytic leukemia | 30 | No | No | Yes (indeterminate etiology) |
| 19 | Renal cell carcinoma | Invasive ductal carcinoma of the breast | 4 | No | No | No |
| 20 | Melanoma | Squamous cell carcinoma of the calf | 2 | No | No | No |
| 21 | Hodgkin's lymphoma | - Basal cell carcinoma - Non-small cell lung cancer | 41, 46 | Yes | Yes | Yes, refractory heart failure from valvular heart disease |
| 22 | Non-Hodgkin's lymphoma | Adenocarcinoma in situ of the cervix | 16 | No | No | No |
| 28 | Hepatocellular carcinoma | Gastrointestinal stromal tumor | 1 | No | No | No |
| 40 | Lung adenocarcinoma in situ | Colon adenocarcinoma | 10 | No | No | No |
| 52 | - Hepatocellular carcinoma - Cholangiocarcinoma | None, both cancers simultaneously diagnosed |  | N/A | No | No |
| 53 | Hodgkin's lymphoma | - Ductal carcinoma in situ of the breast - Basal cell carcinoma | 22, 37 | Yes | No | No |
| 57 | - Invasive ductal carcinoma - Invasive lobular carcinoma | None, both cancers simultaneously diagnosed (one in each breast) | Simultaneous | N/A | No | No |
| 61 | Prostate cancer | Multiple myeloma | 10 | No | No | No |
| 65 | Prostate cancer | Multiple myeloma | 4 | No | No | No |

**Supplemental Table 2:** Characteristics of patients with multiple cancer diagnoses

*Multiple cancer diagnoses*

The 12 patients with multiple cancer diagnoses are profiled in Supplemental Table 2. Two of these patients were deemed to have had secondary cancers due to treatment of their primary cancers. Both these patients had had mantle radiation for Hodgkin’s lymphoma and subsequently developed skin and breast cancers. A third patient developed multiple non-malignant frontal lobe meningiomas from whole body radiation for adrenal neuroblastoma. Two patients each had two separate malignancies diagnosed simultaneously. Only one patient experienced a therapy-related MACCE event. Two patients died, one of an indeterminate etiology and one from refractory heart failure from severe aortic and mitral regurgitation that was not intervened upon due to concomitant advanced metastatic lung cancer (table 8).

*Cardiotoxicity*

Five of the seven events of cardiotoxicity were decompensated heart failure, and occurred in four patients. One patient (#21) developed radiation-associated pericarditis and valvulitis, and was the only patient in the cohort to have had radiation-associated cardiotoxicity. The remaining patient with therapy-related MACCE (#41) developed suspected acute coronary syndrome with chest pain and peak troponin-I level of 0.86 ng/mL four weeks after his first cycle of gemcitabine/ cisplatin. Since this patient’s symptoms resolved within 24 hours of conservative management without dynamic electrocardiographic changes or other high-risk features, additional ischemia evaluation was deferred and the patient successfully tolerated two additional cycles of the same therapy.

Though heart failure was the most common MACCE event, ventricular ejection fraction deteriorated in only one of these five events, in a patient (#3) with metastatic melanoma and possible ICI-associated myocarditis. He had completed a cycle of combination ipilimumab and nivolumab 31 days prior, and presented with decompensated heart failure and newly reduced left ventricular ejection fraction of 36% from 50% noted 1 week prior to initation of ICI therapy. Cardiac troponin-I levels were undetectable and BNP level was elevated at 202 pg/mL, with electrocardiography interpretation limited by chronic ventricular pacing and no cardiac MRI or endomyocardial biopsy being pursued. Since his condition improved with diuresis and initiation of guideline-directed medical therapy without the need for corticosteroids, ipilimumab was discontinued but nivolumab was continued and tremitinib added. His left ventricular ejection fraction improved to 40-45% but never returned to baseline, however he did not have further heart failure episodes on medical therapy. This was also the only ICI-related MACCE event seen and was adjudged a possible myocarditis event due to the lack of troponin or MRI data.

Three patients with therapy-associated heart failure events had significant pre-existing structural cardiac dysfunction. One of these patients (#11) had pre-existing left ventricular dysfunction with ejection fraction of 35% before receiving systemic therapy, while patients #44 and #58 had severe aortic and severe pulmonary regurgitation respectively. All three patients developed acute heart failure in response to intravenous chemotherapy, thought at least in part to be related to intravenous fluid administration. In patient #11, a total of 1.2 L of intravenous fluid was enough to cause acute pulmonary edema, while a second event occurred in the same patient 30 days after initiation of bruiting therapy that resolved with diuresis and cessation of bruiting. Acute decrements in ventricular systolic function on echocardiography were not observed for any of these patients.

The four patients who developed heart failure due to cancer therapy successfully achieved euvolemia with diuretic therapy in the acute setting. They needed either initiation of long-term maintenance oral diuretic therapy or prophylactic diuretic therapy prior to subsequent cycles of systemic therapy. One of these patients (#11) with baseline cardiomyopathy needed scheduled hospitalizations for subsequent chemotherapy cycles. This patient successfully tolerated additional cycles with close monitoring and a customized protocol for administration of intravenous diuresis prior to chemotherapy, based on the amount of intravenous fluid being administered. Two of the four patients were placed on a beta-blocker and renin-angiotensin-aldosterone inhibitor after the heart failure events, while the two others on cardioprotective medications at baseline had these agents continued after the events.
